# Translating Genetic Insights into Molecular Mechanisms in Alzheimer’s Disease: Innovative CRISPR Approaches Targeting Inflammasomes

**DOI:** 10.1002/alz.092397

**Published:** 2025-01-03

**Authors:** Falak Sher

**Affiliations:** ^1^ Columbia University, New York, NY USA

## Abstract

**Background:**

The connection between inflammasomes and Alzheimer’s disease (AD) has garnered significant interest, with emerging evidence suggesting genetic associations and functional implications. Notably, studies have reported the upregulation of inflammasome components like NLRP1, NLRP3, and Caspase‐1 in AD patients. Moreover, genetic polymorphisms in inflammasome‐related genes are linked to increased AD risk. Inflammasome activation in microglia, key immune cells of the brain, contributes to the neuroinflammation characteristic of AD. This backdrop sets the stage for exploring inflammasomes as potential therapeutic targets using advanced genomic tools.

**Methods:**

Addressing the critical need for robust, human‐centric research models, our study developed an optimized protocol to differentiate human induced pluripotent stem cells into microglia (iMG). Validating this model through single‐cell RNA sequencing (scRNA‐Seq), proteomics, and functional assays, we employed a novel CRISPR‐based approach. We conducted comprehensive CRISPR tiling across inflammasome protein coding sequences in iMG cells, coupled with affinity chromatography‐based mass spectrometry and functional guide RNA mapping. This allowed us to investigate the essential subcomplexes within the inflammasome protein family and identify key genetic and protein interactions relevant to AD.

**Results:**

Our CRISPR‐based investigations revealed crucial insights into the inflammasome’s role in AD. We identified specific mutations in NLRP3 that separate its impact on cell health from inflammasome activation. Additionally, our study highlighted critical amino acids in the AD‐associated protein SORL1, offering potential targets for small molecule therapeutics that alter SORL1 function with minimal side effects.

**Conclusion:**

This research marks a significant stride in translating genetic findings into a deeper understanding of molecular mechanisms in AD. By leveraging innovative CRISPR techniques on iMG models, we have elucidated the intricate roles of inflammasome pathways and their interactions with key AD‐associated proteins. Our findings pave the way for novel, targeted therapeutic strategies in Alzheimer’s disease, promising more effective interventions with higher precision.

Our findings offer a generalizable approach to uncover protein or protein‐complex features suitable for rational biochemical targeting

